# In-Field Validation of an Inertial Sensor-Based System for Movement Analysis and Classification in Ski Mountaineering

**DOI:** 10.3390/s18030885

**Published:** 2018-03-16

**Authors:** Jules Gellaerts, Evgeny Bogdanov, Farzin Dadashi, Benoit Mariani

**Affiliations:** 1 UFR Sciences & Montagne (SceM), Département STAPS, Université Savoie Mont-Blanc, Campus Scientifique Technolac, 73376 Le Bourget du Lac, France; 2 Gait Up SA, EPFL Innovation Park, CH-1015 Lausanne, Switzerland; evgeny.bogdanov@crealogix.com (E.B.); farzin.dadashi@gaitup.com (F.D.); benoit.mariani@gaitup.com (B.M.)

**Keywords:** Ski mountaineering, movement analysis, real time measurement, inertial sensors

## Abstract

Ski Mountaineering (SkiMo) is a fast growing sport requiring both endurance and technical skills. It involves different types of locomotion with and without the skis. The aim of this study is to develop and validate in the snowfield a novel inertial-based system for analysing cycle parameters and classifying movement in SkiMo in real-time. The study was divided into two parts, one focused on real-time parameters estimation (cadence, distance from strides, stride duration, stride length, number of strides, slope gradient, and power) and, second, on transition detection (kickturns, skin on, skin off, ski on and off backpack) in order to classify between the different types of locomotion. Experimental protocol involved 16 experienced subjects who performed different SkiMo trials with their own equipment instrumented with a ski-mounted inertial sensor. The results obtained by the algorithm showed precise results with a relative error near 5% on all parameters. The developed system can, therefore, be used by skiers to obtain quantitative training data analysis and real-time feedback in the field. Nevertheless, a deeper validation of this algorithm might be necessary in order to confirm the accuracy on a wider population of subjects with various skill levels.

## 1. Introduction

Ski mountaineering (SkiMo) is a growing sport, on the shortlist for winter Olympics, with an increasing member activity. Yet, there are very few studies on the use of inertial measurement units (IMU) in SkiMo. As Praz et al. [1] suggests, there are few studies that clarify the scientific aspects of SkiMo. SkiMo is generally defined as a discipline that includes at least one period of climbing with “seal skin” under the skis and a downhill period (even if the downhill phase is not present during a climb, for instance, during “vertical race” events). It can also comprise periods of carrying skis on a backpack in steep areas. Typical SkiMo durations range from 30 min to 10 h, with 500 m to 5000 m of altitude gain. In terms of effort, it can be seen as the equivalent of trail running for the winter season, with the additional specificity that it requires technical skills for skiing and transitioning from one locomotion type to another.

In recent years, IMU contributed to the measure and understanding of movement in many fields, such as medicine, sport, and research [2]. Mayagoitia et al. [3] show that kinematic analysis with IMU is equivalent to 3D video for precise measurement in the field, and comparable in laboratory experiments showing high coefficients of multiple correlation in 100% of cases (0.98 ± 0.02). In sports, IMU are used for many reasons, including performance and injury analysis. Gwangjae et al. [4] used IMU sensors to capture turn motions for training in alpine skiing, concluding that data collected from IMU can effectively estimate athlete performance in this sport. In a study by Rawashdeh et al. [5], the authors present an IMU-based detector and classifier to track activity and prevent injuries with real time feedback in volleyball and baseball. The authors report 86% accuracy, moderate for coach and medical staff. The same methods could be used in SkiMo with a specific algorithm. 

Many research studies, and a growing number of products, have been introduced to analyse sports using IMUs, such as swimming, running, and cycling. In swimming, an IMU analysis can guide coaches or researchers in order to measure the swimming biomechanics and consequently help improve swimming analysis, according to Dadashi et al. and Fulton et al. [6,7]. The study by Dadashi et al. validated an IMU algorithm for different phases of the arm stroke with a difference of 0.2 ± 3.9% compared to video capture. The broad application of IMU in sports is confirmed by Harding et al. [8], who developed an algorithm to classify freestyle snowboard rotations with *p* < 0.001, *n* = 216. In running, many studies show IMUs can be an alternative solution to cumbersome video analysis [9] for obtaining movement data in the field, and even obtain acceptable results compared to force platforms when estimating force and power. Setuain et al. [10] estimate running power with IMUs in the field. Xu et al. [11] calculates movement efficiency from IMUs in cycling so coaches can monitor and analyse performance, showing satisfactory results from real-time validation. The authors also developed a smart coaching tool to improve cycling efficiency, which could be applied to other activities, like SkiMo. It is, however, important to note that each application requires a dedicated algorithm to obtain specific parameters from the raw IMU signals. 

Cross-country skiing is the most similar activity to SkiMo. IMUs with dedicated algorithms are already studied in cross-country skiing [12,13,14,15], a discipline that involves similar locomotion to SkiMo during uphill terrain, namely, a gliding phase and a pushing phase (sometimes referred to as thrust) with a cyclical movement. Marsland et al. [12] developed an algorithm in cross-country skiing to identify and classify sub-technics as a tool to analyse and improve performance in training and competition. Recently, Rindal et al. [13] developed an algorithm for classical cross-country skiing biomechanical analysis, their results show an accuracy of 93.9% from the field. 

Fasel et al. [14,16] modified their cross-country skiing algorithm to measure different spatio-temporal parameters in SkiMo, such as cadence, speed, and slope. In this study, different spatio-temporal parameters were calculated in relation to a video reference in the laboratory. The results show an accuracy (precision) of the algorithm on the cycle duration below 3 ms. Accuracy for cycle speed, cycle distance, elevation, and slope gradient were −0.013 m/s (0.032 m/s), −0.027 m (0.018 m), 0.006 m (0.011 m), and 0.40° (0.32°), respectively. Fasel et al. used this system, as well as tools to measure physiological data, while mountaineering in the field, observing associations that improve activity knowledge and allow trainers and athletes to optimize physiology from biomechanics. These analyses were conducted in post-processing and did not allow access to the data in real-time. The authors showed the accuracy of their system in the laboratory setting without further validation in the snowfield condition. Tosi et al. [17] focused on physiological aspects of SkiMo by estimating the energy cost of SkiMo on a slope of 21% grade in the laboratory. In this study, the authors developed a model of mechanical work based on the mass of the subject plus equipment, which can be used as a tool in real conditions in the field. To our knowledge, no study has yet been published about the detection and classification of the different transitions in SkiMo, though it is an essential aspect of SkiMo which can mean the difference between winning or losing a race. In addition to previous methods, a precise analysis of technique during: skin removal, skin attachment, kickturns, or carrying of skis on a backpack, could help reveal the athletic skill level. 

The scope of this study is to develop and validate a novel IMU-based system in the field in SkiMo for real-time movement analyses and classification of the activity with two main objectives:

- Extend, and make available in real-time to coaches and athletes, SkiMo technique parameters to include cycle detection, cadence, glide percentage, stride distance, stride duration, stride length, slope gradient, and power.
- Develop a new algorithm to detect and classify SkiMo transitions between different types of locomotion during the activity.

Our hypothesis was that a novel IMU-based system in SkiMo could analyse all relevant cycle parameters in SkiMo and could classify SkiMo movement in real-time with an accuracy near to 95%. 

## 2. Materials and Methods

### 2.1. Experimental Validation Protocols in the Snow-Field

The protocol was divided into two parts, respectively, for the validation of the SkiMo parameters estimation algorithm, and the validation of the classification performances (Table 1). The two protocols were conducted separately with different subjects and each performed the experiment with their own equipment. All subjects were experienced in SkiMo and all of them signed a consent agreement for their participation.

#### 2.1.1. Parameters Validation Protocol

In this first part, calculated parameters and subjects’ characteristics are presented in Table 1. We collected 25 tests on five different subjects recruited for this study. Subjects were asked to perform standard uphill locomotion in a straight line on a regular slope, in a compact snow, at their self-selected speed for a minimum of 50 strides which were manually counted. The reference position and elevation at starts and end were precisely measured with GPS. 

#### 2.1.2. Transitions Validation Protocol

In the second part, 15 SkiMo tours were collected on 11 different subjects with different duration (39.6 min average, 3.0 min minimum, and 114.15 min maximum). For transition classification validation, subjects were instructed to self-label their activities using a dedicated mobile phone application while performing their own ski tours. Transition validation includes KT, Son, and SOff calculated by the algorithm. KT results are presented in a Bland-Altman graph [18]. To complete this validation, seven thresholds have been tested and are presented with mean-standard deviation in order to have a view on the adjustment of the precision of the algorithm.

The dedicated mobile phone application was developed in order to label the activities during the test. A “labelling” tab allowed the subject to label their movements on the phone during the activity. The phone was securely attached to an arm band for quick and easy access during the activity. For instance, the subject immediately pressed the kick-turn button right after performing a kickturn during the test. The “labelling” tab included all the movements subjects might perform during SkiMo activity. The labelled data was time synchronized and saved in a database for further analysis as a reference for the classifier algorithm. All subjects were familiarized with the testing application through training.

### 2.2. Ski-Mounted IMU Device

**Figure 1:**
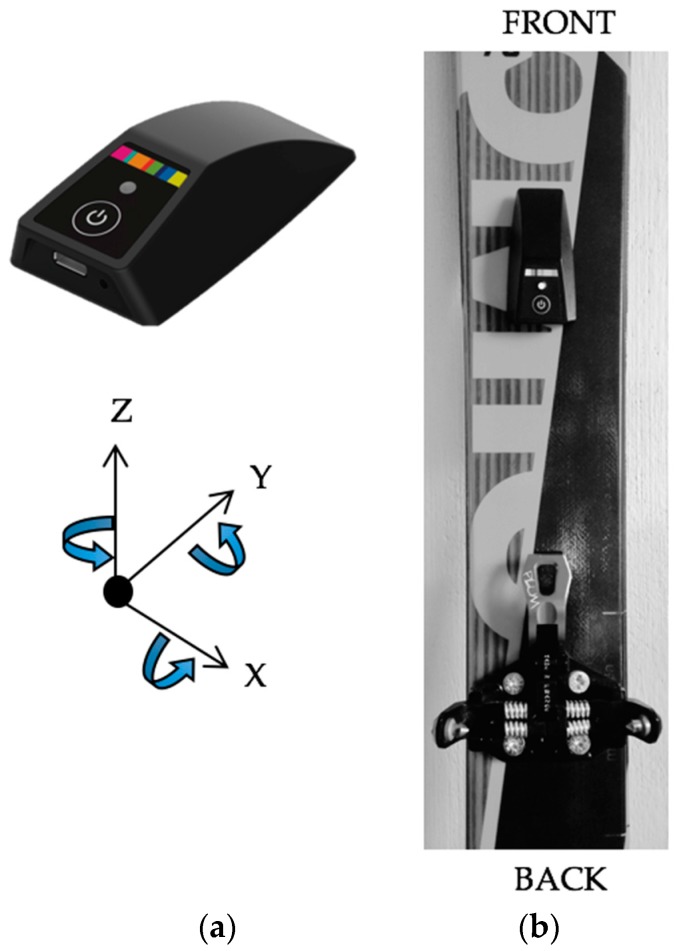
(**a**) Ski-mounted IMU device used for the experiment, including a 3D accelerometer, 3D gyroscope, barometer, and thermometer; and (**b**) device placement on the ski for experimentation.

For the two sections of the experimental protocol, subjects were instrumented with an IMU (Pomoca SA, Denges, Switzerland) composed of a 3D accelerometer, 3D gyroscope, barometric pressure sensor, and thermometer (Figure 1). Dimensions of the device are: 68.1 × 33.6 × 17.7 mm^3^ at 24 g. Accelerometer and gyroscope data were sampled at 128 Hz, and the barometer was sampled at 64 Hz. The system is waterproof for data collection in wet and snowy mountain conditions. Each IMU was composed of the same firmware for all experiments, with raw data stored on the device before being transferred to a computer. The IMU was securely placed on the ski in front of the binding with a magnet (Figure 1b). For all tests, the IMU was manually started from a button press on the device just before the start of the activity.

### 2.3. Algorithm and Reference Parameters Estimation

**Figure 2:**
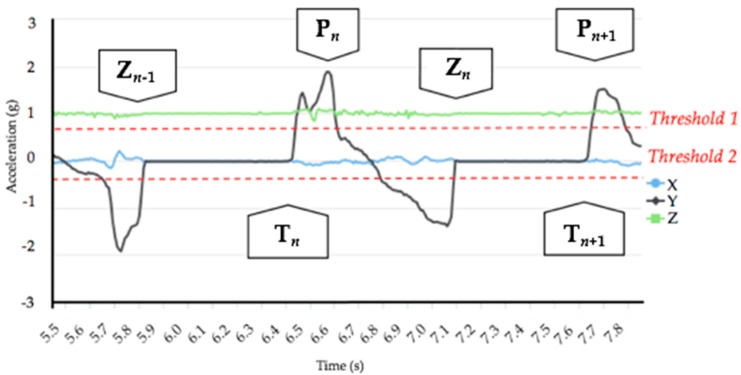
Raw accelerometer signal pattern during SkiMo, with peaks (P*_n_*, P*_n_*_+1_) and periods of motionlessness (Z*_n−1_*, T*_n_*) detected by the algorithm for a given cycle and motion period (T*_n_*, T*_n+1_*).

SkiMo parameters calculated by the algorithm are measured in real time, on a stride-to-stride basis. The data collection follows several definitions presented in this section. The *n*-th cycle is first marked by the algorithm using forward acceleration peaks (P*_n_*_,_ P*_n+1_*), with a dual threshold-crossing method for robust detection of SkiMo pattern features and avoidance of false-positive detection of other movements (Figure 2). A cycle is defined as start of motion at cycle *n* (T*_n_*) to start of motion of cycle *n* + 1 (T*_n_*_+1_). Consequently, the motion period is precisely detected by the algorithm (T*_n_* to Z*_n_*). The algorithm also detects the motionless period of each stride (Z*_n_*, T*_n_*_+1_) by comparing the acceleration norm to 0 g. 

Similar to walking locomotion, a stride consists of two steps, namely, from the sum of right and left legs. For all the parameters computed by the algorithm on a stride-to-stride basis at each cycle *n*, values were finally averaged over the whole trial and compared with reference data.

- **Strides (ST)**

ST_REF_ = total strides manually counted.

ST_ALGO_ = sum of strides detection by the algorithm.

- **Slope gradient (SG) in degrees**

SG was calculated with the elevation measured at the start and end as well as the horizontal distance according to the formula below. Values obtained with this formula were compared with values obtained from the algorithm.

$${\mathrm{SG}}_{\mathrm{REF}}=\mathrm{atan}\frac{H}{\mathrm{HD}}\cdot \frac{180}{\pi }{\;\mathrm{SG}}_{\mathrm{ALGO}}=\mathrm{asin}\left({\mathrm{med}}_{\mathrm{acc}}\right)\cdot \frac{180}{\pi }$$

where H is the vertical distance; HD is the horizontal distance; med_acc_ = median of accelerometer values on *y* axis (forward) during a stride.

- **Stride duration (SD) in seconds**

$${\mathrm{SD}}_{\mathrm{REF}}=\frac{{\mathrm{TD}}_{\mathrm{REF}}}{{\mathrm{ST}}_{\mathrm{REF}}}{\;\mathrm{SD}}_{\mathrm{ALGO}}=T_{n+1}-T_{n}$$

where TD_REF_: total duration (s); and T*_n_*: peak time detected by algorithm for cycle *n*.

- **Cadence (CD) in strides/min**
  $${\mathrm{CD}}_{\mathrm{REF}}=\frac{{\mathrm{TS}}_{\mathrm{REF}}}{{\mathrm{TD}}_{\mathrm{REF}}\cdot 60}{\;\mathrm{CD}}_{\mathrm{ALGO}}=\frac{1\;Stride}{{\mathrm{SD}}_{\mathrm{ALGO}}\cdot 60}$$

- **Stride length (SL) in cm**

$${\mathrm{SL}}_{\mathrm{REF}}=\frac{{\mathrm{Dist}}_{\mathrm{REF}}}{{\mathrm{ST}}_{\mathrm{REF}}}{\;\mathrm{SL}}_{\mathrm{ALGO}}=\iint_{T_{n}}^{Z_{n}}a\;\mathrm{dt}$$

where SL_REF_: stride length for the reference; SL_ALGO_: stride length for the algorithm; Dist_REF_: total distance; and a is the forward (*y*) acceleration signal between start and end motion period T*_n_* and Z*_n_*_._

- **Glide percentage (GP) in %**

**Figure 3:**
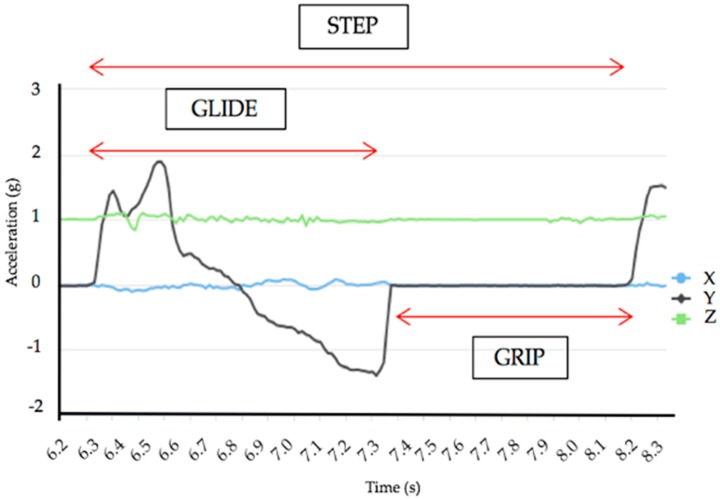
Methods to calculate the glide/grip reference.

To obtain a GP reference, five periods taken at five different times on the raw signal of the accelerometer were analysed and presented as a percentage of steps in order to obtain the grip time and the glide time (Figure 3).

GP_ALGO_ is defined with the motionless detection (grip) and motion detection (glide):

$${\mathrm{GP}}_{\mathrm{ALGO}}=\frac{Z_{n}-T_{n}}{Z_{n}-Z_{n-1}}\cdot 100$$

- **Power (PW) in Watts**

The PW is calculated from the work formula previously calculated by Tosi et al. [17]. The PW was calculated from the formula presented below. In our formula, altitude variation in the potential part of the energy is calculated from the altitude gain or vertical distance (H) and not by the angle of the slope. It does not include the friction and aerodynamics forces, but takes into account the mass of the subject (Ms), the mass of his equipment (Me), and the mass of a ski, a boot, and binding (Mb):

$${\mathrm{PP}}_{\mathrm{REF}}=\frac{\left(\mathrm{Ms}+\mathrm{Me}+\mathrm{Mb}\right)\cdot g\cdot H_{\mathrm{REF}}}{{\mathrm{TD}}_{\mathrm{REF}}}\;{\mathrm{PP}}_{\mathrm{ALGO}}=\frac{\left(\mathrm{Ms}+\mathrm{Me}+\mathrm{Mb}\right)\cdot g\cdot H_{\mathrm{ALGO}}}{{\mathrm{SD}}_{\mathrm{ALGO}}}$$

$${\mathrm{PK}}_{\mathrm{REF}}=\frac{16\cdot \mathrm{Mb}\cdot V_{\mathrm{REF}}^{2}}{{\mathrm{TD}}_{\mathrm{REF}}}{\;\mathrm{PK}}_{\mathrm{ALGO}}=\frac{16\cdot \mathrm{Mb}\cdot V_{\mathrm{ALGO}}^{2}}{{\mathrm{SD}}_{\mathrm{ALGO}}}$$

$${\mathrm{PW}}_{\mathrm{REF}}={\mathrm{PP}}_{\mathrm{REF}}+{\mathrm{PK}}_{\mathrm{REF}}{\;\mathrm{PW}}_{\mathrm{ALGO}}={\mathrm{PP}}_{\mathrm{ALGO}}+{\mathrm{PK}}_{\mathrm{ALGO}}$$

where PP: potential power; PK: kinetic power; H_REF_: elevation gain between the start and the end of the trial; V_REF_: speed average during activity measured by hypotenuse distance divided by time; H_ALGO_: altitude gain variation measured by the barometer for each stride; and V_ALGO_: speed calculated by stride length divided by time.

Results for PW_ALGO_ are obtained with the mean of each stride.

- **Distance from strides**

$${\mathrm{DFS}}_{\mathrm{REF}}={\mathrm{Dist}}_{\mathrm{REF}}{\;\mathrm{DFS}}_{\mathrm{ALGO}}=\sum^{\text{​}}{\mathrm{SL}}_{\mathrm{ALGO}}$$

Algorithm and Reference Definition of Transition Classes.

**Figure 4:**
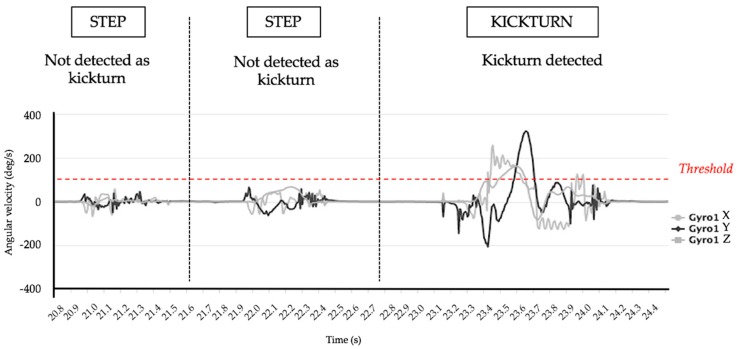
Kickturn definition threshold.

The algorithm detects the kickturn (KT) during SkiMo based on the gyroscope signal. During the activity, and contrary to standard uphill forward locomotion, a KT can be characterized by a positive or negative rotation along the *z* gyroscope axis (Figure 1a). Our results show that, by only applying a threshold to the gyroscope value (Figure 4), all KT during an activity are detected. Moreover, we introduced a constraint to avoid false positive KT detection by accepting a KT only during uphill skiing.

Skins On (SOn) transitions are defined by a vertical position of the ski between a period of downhill and uphill or at the beginning of the activity. Skins Off (SOff) transitions were defined by a motionless period of a minimum of six seconds between an ascent and a downhill. Finally, to detect backpack (BP) transitions, the conditions were that the ski must be close to the vertical while in motion and with a small altitude gradient.

### 2.4. Statistical Analysis

**Figure 5:**
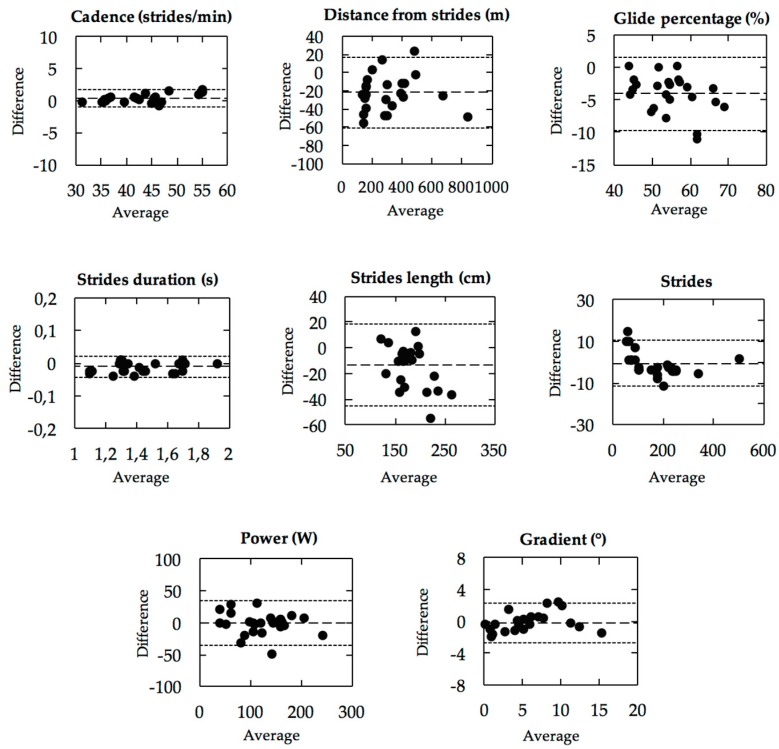
Bland-Altman plot with mean (*x*-axis) and difference between (*y*-axis) algorithm and reference for all parameters. Limits of agreement are specified as the average difference (dashed line) ± 1.96 standard deviation of the difference (dotted line).

**Figure 6:**
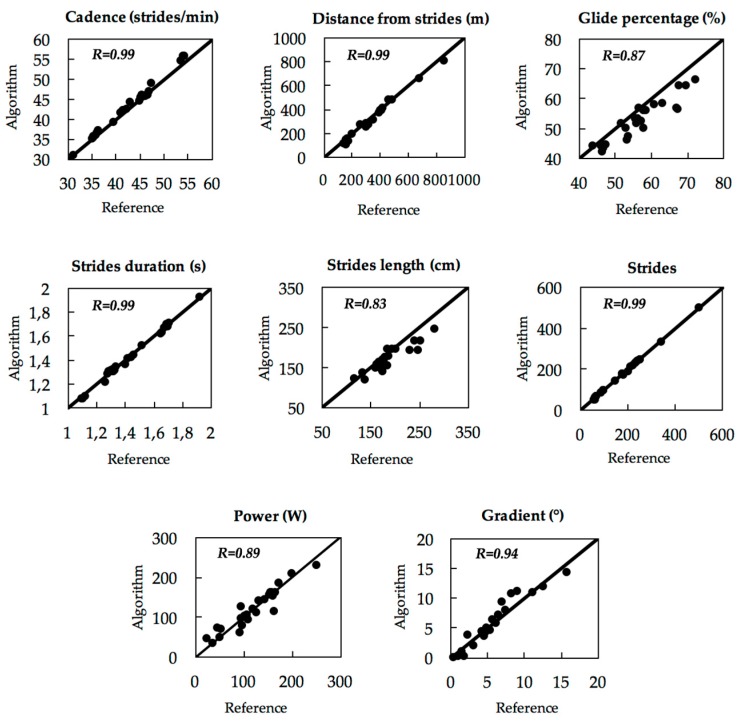
Linear correlation with reference for all parameters (*x*-axis) and algorithm (*y*-axis) and *y* = *x* (bold); *n* = 25 trials obtained from five subjects.

Results obtained from the algorithm and the reference were analysed using the Bland-Altman method [19] and their association was quantified with Pearson’s r correlation (Figure 5 and Figure 6). In addition, results are presented with mean absolute difference and mean relative difference with standard deviation (Table 2).

Results for transition classification were analysed using the Bland-Altman method and results are presented as mean ± standard deviation. In addition, the specificity-sensitivity (SE-SP) values for kickturns detection were analysed for different thresholds values (Table 3). Statistical analysis was performed using XLstat software (Addinsoft, France).

## 3. Results

### 3.1. Parameter Estimation

Parameter validity is presented in Figure 5 with the Bland-Altman method. The results are also presented in Table 2 and show an accuracy ± precision of 0.6 ± 1.5% for CD, −9.7 ± 9.6% for DFS,−6.9 ± 4.5% for GP, −0.8 ± 1.2% for SD, −6.5 ± 7.7% for SL, −4.1 ± 5.8% for ST, −4.9 ± 26.2% for PW, and 1.1 ± 0.7° for SG. The SG was not presented in percent because the values are sometimes close to, or equal to, 0. On the other hand, correlation curves are presented in Figure 6, with method accuracy of *R*^2^ = 0.99 for CD, *R*^2^ = 0.99 for DFS, *R*^2^ = 0.87 for GP, *R*^2^ = 0.99 for SD, *R*^2^ = 0.83 for SL, *R*^2^ = 0.99 for ST, *R*^2^ = 0.89 for PW, and *R*^2^ = 0.94 for SG.

All results for the movement parameters are presented in Table 2. To facilitate interpretation, we can specify that a positive mean relative difference corresponds to an overestimation of the algorithm compared to the reference. On the other hand, a negative value corresponds to an underestimation of the algorithm. 

### 3.2. Transitions

#### 3.2.1. Kickturns Detection

**Figure 7:**
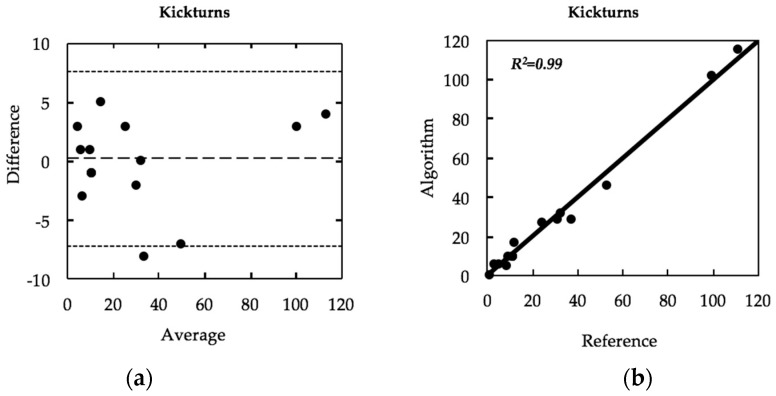
Bland-Altman (**a**) and linear correlation (**b**) for KT with a 95°/s threshold. Difference is calculated by algorithm—reference (positive value = real value overestimated by algorithm (15 trials by 11 subjects).

KT results are presented in Figure 7. Figure 7a shows an average difference of 0.2 kickturns with a limit agreement set at [−7.17, 7.57]. Results do not show any visible bias with the number of kickturns performed during an activity. Moreover, Figure 7b does not confirm any random error of kickturns *R*^2^ = 0.99. Note that if the two values approaching 100 kickturns are removed the value becomes *R*^2^ = 0.95.

Different thresholds were tested for the detection of kickturns, with results in Table 3. We can observe the absolute mean difference for each threshold. Results show an increased efficiency of the algorithm on values between 90 and 100°/s with an optimum accuracy of −0.2 at a threshold value of 95°/s.

SE-SP values are also presented in Table 3 and show a 95% SP for an SE of 79% for a threshold set at 95°/s. At 50°/s and 150°/s SE-SP values are, respectively, 97% SE, 69% SP and 78% SE, 88% SP.

#### 3.2.2. Other Transitions Detection and Classification

**Figure 8:**
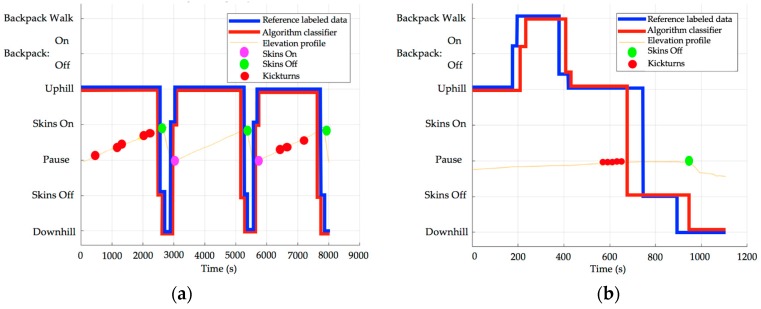
(**a**) Comparison between reference-labelled data and algorithm classifier with a subject who performed a tour: uphill, skins off, downhill, skins on, uphill, skins off, downhill; *R* = 0.98; (**b**) Comparison between reference-labelled data and algorithm classifier with a subject who performed a tour: uphill, backpack on, backpack walk, backpack off, uphill, skin off, downhill; *R* = 0.90.

The number of other transitions, namely, SOn, SOff, and BP, were compared to the reference labelling and are presented with absolute difference in Table 4. Two representative example are shown in Figure 8a,b with the time on the *x* axis and class of activity on the *y* axis.

## 4. Discussion

The aim of this study is to validate, in the snowfield, a novel IMU-based system for analysing movement parameters and classifying SkiMo data in real-time. Two main aspects have been studied, making available in real-time to coaches and athletes, SkiMo technique parameters and developing a new algorithm to detect and classify SkiMo transitions between different types of locomotion during the activity.

In the results, CD values have an accuracy ± precision of 0.6 ± 1.5% for low, moderate, and high cadence. Praz et al. measured CD in a previous study to evaluate optimal SkiMo slopes and speed in the field [15]. However, this finding was not displayed in real-time in the field. Consequently, our work could improve and accelerate the analysis for future researchers. Thus, our results might be suitable for race tourers, sometimes referred to as speed-tourers, as well as pure recreational tourers, to precisely analyse their SkiMo cadence.

Our results for SD and SL show values close to 0% and 5%, respectively, confirming the robustness of our algorithm for computing these parameters. Indeed, even though some SL values show an error of approximately 30 cm between 150 and 200 cm strides, the majority give less than 10 cm error between 150 and 200 cm SL, and confirm that the algorithm gives precise values. Fasel [16] researched an algorithm designed for diagonal stride, adapted for SkiMo, and validated on a treadmill with SD and SL algorithms showing an accuracy (precision) of 0.0 ± 0.2% and −1.6 ± 1.0%. Compared to Fasel’s laboratory results, the accuracy of our algorithm confirms the robustness of step duration measurement in the field. Moreover, increased SL values might be associated with the field condition.

PW was calculated from the formula of Tosi et al. [17]. This parameter was validated by comparing our algorithm results with the reference. The results show an average difference of 12.1 ± 12.6 W. Although such a precision is not comparable with real power measured in a laboratory setting, it allows the monitoring of intra-subject power changes. PW calculated from the model of Tosi et al. gives a first approximation of the mechanical PW developed in SkiMo. With the first results, PW has a strong potential as a training and racing measure for athletes and trainers, for instance to define adequate training zones and time to exhaustion, as in cycling [19]. Further studies, with more precise reference systems, are required to refine the current power estimation model. Moreover, in a study by Breitschädel et al. [20], cross-country skiing tests were performed using an IMU attached to the ski. These authors apply an algorithm for acceleration loss to the skier during a gliding test. The parameters calculated by their algorithm were used by the researchers to bring out specific data, in particular the coefficient of friction at the ski-snow interface. This algorithm provides a variety of motion related to mechanical parameters. Therefore, researchers could conduct in-depth research by coupling an energy analysis device to calculate the energy cost of the skier without resorting to consistent data processing.

SG results show 1.1° ± 0.7°, which is in the range of error for using inertial sensors as inclinometers as Fasel [16] showed on a treadmill with an accuracy (precision) of 0.4° ± 0.3°. Compared to Fasel, SG results decreased in the field, but we posit this parameter is valid and gives a very high-resolution measure of the instantaneous slope gradient during SkiMo. As examples, this may be used to train on a constant slope, or to measure the slope angle for avalanche safety. 

GP results show an error variation between 1.6% and −9.7% with a slight algorithm bias, in the form of a slight underestimation. The average inter-subject error is 4.1 ± 2.9%. The GP has an average error less than 5%. A few recordings with error around 10% can be explained by the manual reference, which is also prone to error. In particular, since the GP reference was manually calculated, the signal sometimes varied, thus influencing the mean, even when the subject was instructed to perform the test with a regular movement for regular glide. Glide was precisely calculated by Fasel and used by Praz [15,16]. Compared to Fasel, GP includes the moving phase in addition to the gliding phase. The increase of GP indicates a longer glide phase compared to the grip phase. Consequently, GP calculation could present a new method to estimate movement efficiency in SkiMo.

Obviously, step number is calculated by this algorithm. Our results show an underestimation of 4.1 ± 5.8%, on average, for distances between 200 and 1000 m. This parameter tends to be slightly lower than the reference, but calculated values seem close to reality for step counts up to 1000 m in our results. No study has been published on cycle detection in the field with varied snow conditions, which could be an interesting study to improve algorithm accuracy in the future.

In addition, DFS results were calculated and show low error over distances between 200 and 1000 m. This parameter shows it is possible to calculate the approximate distance without GPS. This feature allows recording and analysis of long races and training sessions, several hours beyond the battery capacity of GPS-based devices, which we find is typically a few hours, based on a brief review of common watches and smartphones available on the market. 

Several limitations of our algorithm validation procedures are now discussed. We included a limited number of subjects in our study, and the effect of repeated measures of the same person may have influenced our results. The subjects performed free and unsupervised tests with manually-reported reference values, which are subject to human error and might have influenced our results. Moreover, the distance of each test could be increased in future studies in order to control and validate the algorithm over distances greater than 1000 m.

The second part of our results concerned transition detection of KT, SOn, SOff, and BP. Our results were calculated by the algorithm and compared to reference labelling by subjects, who used the “labelling” tab presented earlier in this study. Our results show an average error of −0.2 ± 3.8 KT. We tested different thresholds to determine 95°/s was optimal for the detection of KT. With our robust kickturn detection in place, a future step is in-depth characterization of kickturns by computing the duration or kinematics. This analysis could create interesting new approaches to training and teaching. For a total of 40 SOn, 59 SOff, and 38 BP, the results show a mean difference –0.2 ± 0.6, −0.1 ± 0.4, and −0.1 ± 0.5, respectively. Similarly, Rindal et al. [13] achieved automatic classification of classical cross-country skiing movements with an accuracy of 93.9%, with fewer transitions result for SkiMo due to an easier identification of movement. Their work related to automatic classification includes different, and easier to detect, movements, with the same objective to “provide novel insight into physiological and biomechanical aspects valuable to coaches, athletes, and researchers”. Finally, our algorithm proves to be very precise in the classification of SkiMo movements, allowing seamless and automatic classification of activity during tours. 

Our study is the first to introduce activity classification in SkiMo. Various practical applications of the presented system emerged from this study. The algorithm output could be interesting for various groups of ski tourers, from recreational tourers who might look for automatic quantification of their tours and basic safety measures, such as slope, to racing tourers, who are interested in improving the level of technique and performance. As a tool for researchers in sport-sciences, the system can be used to strengthen the understanding of the links between movement, technique, and physiology. The presented system facilitates quantitative data collection in the field, and makes it possible to accelerate and improve the activity analysis, with results available during the activity and immediately after. 

## 5. Conclusions

The aim of this study is to develop and validate in the snowfield a novel inertial-based system for analysing cycle parameters and classifying movement in SkiMo in real-time. The presented algorithm extends the list of parameters which can be extracted in real-time on a stride-to-stride basis, and introduces, for the first time, an automatic SkiMo activity classification method based on a robust detection of transitions. Our results prove the algorithm is acceptable, with comparable accuracy to state-of-the art studies on inertial sensor-based systems introduced in other sports. Since the system is lightweight and easy-to-use, it is suitable for various practical applications in training, safety, and research. Yet, it is necessary to further perform more complete and precise tests in order to refine the methods, notably on power estimation, and validate the system on a larger number of subjects.

## Figures and Tables

**Table 1:** Parameters (A) and transitions classification (B) analysed in the study with abbreviations. Participant characteristics are also presented (mean ± standard deviation).

Protocol	(A) Parameters Estimation	(B) Transitions Classification
Number of subjects	5	11
Age	47.4 (±12.5)	40.1 (±13.1)
Weight (kg)	71.7 (±5.6)	72.6 (±5.1)
Height (cm)	179 (±4.7)	180.4 (±5.0)
Output of the algorithm:	Cadence (CD) Distance from strides (DFS) Glide percentage (GP) Power (PW) Stride duration (SD) Stride length (SL) Stride (ST) Slope gradient (SG)	Kickturns (KT) Skin On (SOn) Skin Off (SOff) Backpack (BP)

**Table 2:** Mean absolute difference and relative difference between algorithm and reference for all parameters. Difference is calculated by algorithm—reference. Positive values indicate an overestimation of the real value by the algorithm; 25 trials with five subjects.

*n* = 25 (5 Subjects)	Algorithm (±std)	Reference (±std)	Mean Absolute Difference (±std)	Mean Relative Difference% (±std)
Cadence (steps/min)	86.7 (±13.2)	86.1 (±12.5)	1.1 (±1.0)	0.6 (±1.5)
Distance from Strides (m)	287.3 (±178.0)	303.8 (±178.0)	25.5 (±14.8)	−9.7 (±9.6)
Glide percentage (%)	52.9 (±6.7)	56.9 (±7.8)	4.1 (±2.9)	−6.9 (±4.5)
Stride duration (s)	1.41 (±0.22)	1.42 (±0.21)	0.01 (±0.01)	−0.83 (±1.2)
Stride length (cm)	171.9 (±30.1)	185.4 (±38.0)	15.6 (±14.1)	−6.5 (±7.7)
Strides	165.0 (±106.3)	169.2 (±105.2)	4.4 (±3.7)	−4.1 (±5.8)
Power (W)	119.7 (±50.8)	120.2 (±54.2)	12.1 (±12.6)	−4.9 (±26.2)
Slope gradient (°)	4.6 (±5.0)	4.9 (±4.3)	1.1 (±0.7)	−11.1 (±86.1)

**Table 3:** Mean absolute difference between reference and algorithm with sensibility (SE)—specificity (SP) and 95% confidence interval (CI) for 15 trials. Difference was calculated by algorithm—reference for each subject for all thresholds.

Kickturn Threshold (°/s)	Accuracy (Precision)	Sensibility (SE)% + 95% CI	Specificity (SP)% + 95% CI
50	21.7 (±19.7)	97% [95; 98]	69% [66; 71]
70	5.1 (±7.0)	96% [94; 98]	76% [72; 79]
90	0.5 (±3.6)	95% [92; 96]	78% [72; 82]
95	−0.2 (±3.8)	95% [93; 97]	79% [73; 84]
100	−0.8 (±3.5)	95% [92; 96]	78% [72; 83]
110	−1.7 (±3.0)	93% [90; 95]	78% [70; 84]
130	−4.3 (±5.3)	86% [82; 89]	77% [67; 85]
150	−7.2 (±7.7)	78% [74; 82]	88% [76; 95]

**Table 4:** Absolute difference between algorithm and reference for movement type (KT, SOn, SOff, BPOn/off).

*n* = 15 (11 Subjects)	Algorithm	Reference	Difference A–R (std)
SkinOn (total: 40)	0.8 (±1.0)	0.9 (±1.1)	−0.2 (±0.6)
SkinOff (total: 59)	1.8 (±1.0)	1.9 (±1.0)	−0.1 (±0.4)
Backpack On/Off(total: 38)	2.3 (±1.9)	2.4 (±2.1)	−0.1 (±0.5)

## Abbreviations

IMU	Inertial Measurement Unit
CD	Cadence (steps/min)
DFS	Distance from strides (m)
GP	Glide percentage (%)
PW	Power (W)
SD	Stride duration (s)
SL	Stride length (cm)
ST	Stride
SG	Slope Gradient (°)
KT	Kickturn
SOn	Skin on
SOff	Skin off
BP	Backpack
SE	Sensitivity (%)
SP	Specificity (%)
